# SKF96365 modulates activity of CatSper channels in human sperm

**DOI:** 10.1093/molehr/gaad015

**Published:** 2023-04-27

**Authors:** Elis Torrezan-Nitao, Sean G Brown, Linda Lefievre, Jennifer Morris, Joao Correia, Claire V Harper, Stephen Publicover

**Affiliations:** School of Biosciences, University of Birmingham, Birmingham, UK; School of Applied Sciences, Division of Health Sciences, Abertay University, Dundee, UK; Institute of Clinical Sciences, School of Biomedical Sciences, University of Birmingham, Birmingham, UK; School of Biosciences, University of Birmingham, Birmingham, UK; School of Biosciences, University of Birmingham, Birmingham, UK; Institute of Metabolism and Systems Research, School of Biomedical Sciences, University of Birmingham, Birmingham, UK; Department of Biology, Faculty of Arts and Sciences, Edge Hill University, Ormskirk, Lancashire, UK; School of Biosciences, University of Birmingham, Birmingham, UK

**Keywords:** human sperm, SKF96365, CatSper, progesterone, calcium, calcium oscillations, patch clamp, cation channel of sperm

## Abstract

Exposure of human sperm to progesterone (P4) activates cation channel of sperm (CatSper) channels, inducing an intracellular Ca^2+^ concentration ([Ca^2+^]_i_) transient followed by repetitive [Ca^2+^]_i_ activity (oscillations), which are believed to be functionally important. We investigated the potential significance of store-operated Ca^2+^-entry in these oscillations using the inhibitor SKF96365 (30 µM; SKF). Following pre-treatment of human sperm with 3 µM P4, exposure to SKF doubled the proportion of oscillating cells (*P* = 0.00004). In non-pre-treated cells, SKF had an effect similar to P4, inducing a [Ca^2+^]_i_ transient in >80% of cells which was followed by oscillations in ≈50% of cells. The CatSper blocker RU1968 (11 µM) inhibited the SKF-induced [Ca^2+^]_i_ increase and reversibly arrested [Ca^2+^]_i_ oscillations. Using whole-cell patch clamp, we observed that SKF enhanced CatSper currents by 100% within 30 s, but amplitude then decayed to levels below control over the next minute. When cells were stimulated with P4, CatSper currents were stably increased (by 200%). Application of SKF then returned current amplitude to control level or less. When sperm were prepared in medium lacking bovine serum albumin (BSA), both P4 and SKF induced a [Ca^2+^]_i_ transient in >95% of cells but the ability of SKF to induce oscillations was greatly reduced (*P* = 0.0009). We conclude that SKF, similar to a range of small organic molecules, activates CatSper channels, but that a secondary blocking action also occurs, which was detected only during patch-clamp recording. The failure of SKF to induce oscillations when cells were prepared without BSA emphasizes that the drug does not fully mimic the actions of P4.

## Introduction

Intracellular Ca^2+^ concentration ([Ca^2+^]_i_) signalling is crucial to sperm function, being a key regulator of motility, acrosome reaction, and capacitation (Darszon *et al.*, 2005; Publicover *et al.*, 2008; Prajapati *et al.*, 2022). A number of stimuli, including zona pellucida (ZP), are believed to regulate human sperm function by this pathway, the best characterized being the steroid progesterone (P4), which is secreted by the cells of the cumulus and is present throughout the female tract (Correia *et al.*, 2007). P4 acts on human sperm by activation of the sperm-specific Ca^2+^-permeable channel CatSper (Lishko *et al.*, 2011; Strunker *et al.*, 2011), which, in human sperm and those of many other species, is the principal mechanism of Ca^2+^ mobilization (Lishko *et al.*, 2012; Lishko and Mannowetz, 2018).

In addition to CatSper, Ca^2+^ stores and capacitative (store-operated) Ca^2+^ entry (CCE) are implicated in sperm [Ca^2+^]_i_ signalling (Costello *et al.*, 2009; Correia *et al.*, 2015). CCE may be triggered by CatSper-mediated elevation of [Ca^2+^]_i_, serving to amplify, propagate and/or prolong the [Ca^2+^]_i_ signal (Alasmari *et al.*, 2013; Correia *et al.*, 2015). No specific pharmacological agents are available for blockade of CCE, but 10–30 µM SKF96365 (SKF) is a known and well-characterized antagonist of CCE both in cell lines and in a range of acutely cultured cell types (Labelle *et al.*, 2007; Ng *et al.*, 2008, 2010; Li *et al.*, 2010; Sabourin *et al.*, 2016). SKF has also been shown to inhibit calcium-release activated Ca^2+^ (CRAC) current (a form of CCE; Parekh and Putney, 2005) in cells where CRAC currents occur naturally and also in cells transfected with Orai, a pore forming channel which is involved in CRAC and other CCE channels (Prakriya and Lewis, 2002; Yeromin *et al.*, 2006; Varnai *et al.*, 2009).

SKF has been used as a tool to investigate the participation of CCE in sperm functions. Treatment with SKF inhibited acrosome reaction in sea urchin sperm (Hirohashi and Vacquier, 2003) and suppressed the increase in [Ca^2+^]_i_ induced by mitochondrial inhibitors (Ardon *et al.*, 2009). In mouse sperm, SKF inhibited Ca^2+^ influx and acrosome reaction induced by maitotoxin (Trevino *et al.*, 2006) and suppressed motility (Ru *et al.*, 2015). In human sperm, SKF has been reported to suppress motility (Castellano *et al.*, 2003) to inhibit acrosomal swelling (believed to be a key stage in acrosome reaction) induced by P4 and by thapsigargin, which mobilizes stored Ca^2+^; Sosa *et al.*, 2016), and to inhibit acrosome reaction induced by recombinant ZP3 (Jose *et al.*, 2010).

Human sperm stimulated with P4 generate an ‘instantaneous’ CatSper-mediated Ca^2+^ transient, which is followed by a plateau of increased [Ca^2+^]_i_ upon which [Ca^2+^]_i_ oscillations are often superimposed (Harper *et al.*, 2004; Kirkman-Brown *et al.*, 2004; Aitken and McLaughlin, 2007; Sanchez-Cardenas *et al.*, 2014; Mata-Martinez *et al.*, 2018). Pharmacological manipulations suggest that this latter part of the [Ca^2+^]_i_ response may involve secondary release of stored Ca^2+^ (Harper *et al.*, 2004; Servin-Vences *et al.*, 2012; Correia *et al.*, 2015; Mata-Martinez *et al.*, 2018), and CCE (Lefièvre *et al.*, 2012; Mata-Martinez *et al.*, 2018). We therefore investigated the effect of SKF on both P4-induced and basal [Ca^2+^]_i_ signalling in human sperm. SKF might be expected to suppress [Ca^2+^]_i_ signalling in human sperm. Instead, we observed a dose-dependent increase in [Ca^2+^]_i_ upon SKF-treatment. This effect of SKF resembled the well-characterized action of P4 on human sperm and further investigation confirmed that the drug modulates activity of CatSper channels.

## Materials and methods

### Materials

All chemicals were obtained from Sigma-Aldrich (Poole, UK) except fluo4-AM (acetoxymethylester), which was from Thermo Fisher Scientific, Swindon, UK. As the observed effect of SKF was unexpected, we tested the drug from four different suppliers [Calbiochem/Merck (UK), Tocris (Abingdon, UK), Abcam (Cambridge, UK), and Sigma-Aldrich]. Similar results were obtained in every case. Fluo4-AM was prepared in dimethylsulphoxide (DMSO) containing 20% Pluronic F-127 (Thermo Fisher). P4 and RU1968 were dissolved in DMSO at 10 mM and diluted in supplemented Earle’s balanced salt solution (sEBBS) prior to use. The concentration of DMSO in imaging experiments was 0.03–0.3%. RU1968 was a kind gift of Dr Timo Strünker, Centre of Reproductive Medicine and Andrology, Münster, Germany.

### Salines

The standard incubation medium used in this study was sEBSS, containing NaCl (90 mM), KCl (5.4 mM), CaCl_2_ (1.8 mM), MgCl_2_ (1 mM), glucose (5.5 mM), NaHCO_3_ (25 mM), Na pyruvate (2.5 mM), Na lactate (19 mM), MgSO_4_ (0.81 mM), HEPES (15 mM), and 0.3% bovine serum albumin (BSA). The pH was adjusted to 7.4 with NaOH and osmolarity was then adjusted to 291–294 mOsm as necessary by adding NaCl.

For patch clamp, the standard bath solution contained NaCl (135 mM), KCl (5 mM), CaCl_2_ (2 mM), MgSO_4_ (1 mM), HEPES (20 mM), Glucose (5 mM), Na pyruvate (1 mM), Lactic acid (10 mM), pH adjusted to 7.4 with NaOH. The Cs^+^-based bath solution contained Cs-methanesulphonate (140 mM), HEPES (40 mM), EGTA (3 mM), EDTA (3 mM), pH adjusted to 7.4 with CsOH. Pipette solution contained Cs-methanesulphonate (130 mM), HEPES (40 mM), Tris–HCl (1 mM), EGTA (3 mM), EDTA (3 mM), pH adjusted to 7.4 with CsOH.

### Selection and preparation of spermatozoa

Written consent was obtained from donors in accordance with the Human Fertilisation and Embryology Authority (HFEA) Code of Practice (versions 7 and 8) under local ethical approval (South Birmingham Local Ethical Committee (Ref: 2003/239), and University of Birmingham (ERN 07-009 and ERN-12-0570)). Samples were obtained by masturbation after 2–3 days sexual abstinence. After liquefaction (30 min), sperm were swum up into sEBSS (60 min), adjusted to a maximum of ≈6×10^6^/ml and left to capacitate (36 °C, 5.5% CO_2_) for 5 h.

### Collection and analysis of imaging data

Imaging was carried out as described (Nash *et al.*, 2010). Briefly, after adjusting sperm concentration to 1.5×10^6^/ml, the cell suspension was divided into aliquots of 200 μl and incubated with fluo4-AM (5 μM) for 30 min (36 °C, 5.5% CO_2_). Cells were then transferred to a perfusable imaging chamber, the base of which was a coverslip coated with 0.001% poly-D-lysine and incubated for an additional 5 min to allow cells to settle. The chamber was installed on the stage of an inverted fluorescence microscope (Nikon TE300) and perfused with sEBSS to remove unattached cells and excess dye. All experiments were performed at 25 °C in a continuous flow of sEBSS, with a perfusion rate of 0.6 ml/min. Fluorescence excitation was at 470–480 nm (Zeiss or Cairn 75 W xenon source with filter or OptoLED, Cairn, UK) and emission at 520 nm. Images were captured at 0.1–0.2 Hz using a 40× or 60× oil-immersion objective and a Hamamatsu Orca 1 or Q Imaging Rolera-XR CCD camera or an Andor Ixon 897 EMCCD camera controlled by iQ software (Andor Technology, Belfast, UK). Stimuli were applied to the cells by inclusion in the perfusing medium.

Analysis of images and background correction was done using iQ software (Andor Technology). Regions of interest were drawn around the posterior head/neck of each cell and the background subtracted. Average intensity was obtained for each area, imported into Microsoft Excel and normalized by calculating the percentage change in fluorescence (Δ*F*) using the equation: where Δ*F* is the percentage change in fluorescence intensity at time *t*, *F* is fluorescence intensity at time *t* and *F*rest is the mean of ≥10 determinations of *F* during the control period before application of any stimulus. For comparison of the amplitudes of P4 and SKF-induced [Ca^2+^]_i_ signals, a mean response of all cells was calculated for each experiment. SKF and P4-induced transient amplitudes were calculated as the increment in Δ*F* (the difference between the Δ*F* values at the signal peak and immediately before onset of the signal) and statistical tests were carried out using these values, unless stated otherwise.


ΔF=[(F−Frest)/Frest]×100%

Frequency of repetitive [Ca^2+^]_i_ activity (oscillations) induced by P4 and SKF stimulation was analyzed manually by counting the number of [Ca^2+^]_i_ spikes and dividing by time. Background [Ca^2+^]_i_ noise or ‘ripples’ (typically <20% change in fluorescence of fluo4) were not considered oscillations (Torrezan-Nitao *et al.*, 2021).

### Fluorimetry of [Ca^2+^]_i_ and pHi

Dose-dependences of [Ca^2+^]_i_ signals induced by P4 and SKF were assessed in fluo4-loaded cells suspended in sEBSS (6 million/ml), using a FLUOstar microplate reader (BMG Labtech, Offenburg, Germany) as described previously (Achikanu *et al.*, 2018). In each experiment, four to six doses of P4 or SKF and a vehicle control (DMSO; at a concentration equivalent to that present in the highest dose used: 0.6%) were applied simultaneously. Dose–response data were fitted with a four parameter logistic regression model (*Y* = min+(max−min)/1+(*X*/EC_50_)×Hill coefficient) using https://mycurvefit.com/. pHi was assessed in 2′,7′-bis-(2-carboxyethyl)-5-(and-6)-carboxyfluorescein, acetoxymethyl ester (BCECF, AM)-loaded cells using a FLUOstar microplate reader, as described in Achikanu *et al.* (2018). Emission at 530 nm was measured in response to alternating excitation at 440 and 490 nm and was then used to calculate a 490/440 ratio.

### Electrophysiology

Capacitated cells were re-suspended in standard bath solution and allowed to adhere to glass coverslips that were transferred to an inverted microscope where they were superfused with standard bath solution Whole cell CatSper currents were then recorded using Cs^+^-based divalent-free pipette and bath solutions (Lishko *et al.*, 2011). Currents were evoked by a ramp protocol (−80 to 80 mV over 1 s), which was repeated at 2 s intervals to allow continuous monitoring of current amplitude. Membrane potential was held at 0 mV between ramps. Data were sampled at 2 kHz, filtered at 1 kHz.

### Statistics

Data were assessed for normality using the Anderson–Darling test and tested accordingly. Percentage data were transformed using the arcsine square root conversion (Sokal and Rohlf, 1981) before statistical analysis to allow application of parametric tests. Chi-square test was used for categorical variables (with adjustment for multiple testing as appropriate). The Student’s *t*-test (paired or independent), Mann–Whitney or Wilcoxon test, with adjustment for multiple testing as appropriate, were used for continuous variables. Data are presented as mean±SEM. Statistical analysis was carried out using Minitab 18 (Minitab, PA, USA).

## Results

### SKF96365 does not inhibit [Ca^2+^]_i_ oscillations

**Figure 1.**
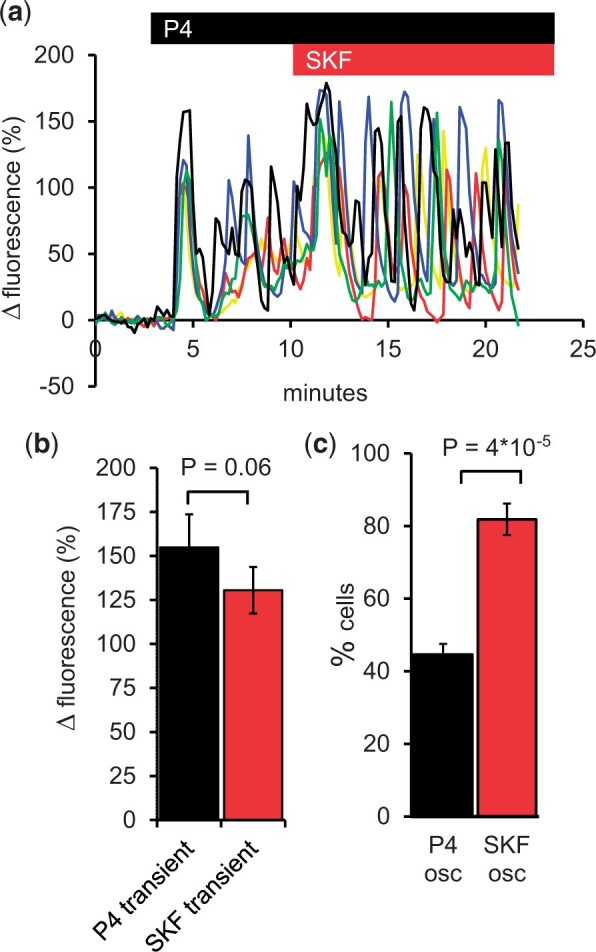
**Progesterone-induced [Ca^2+^]_i_ oscillations in human sperm are not inhibited by 30 µM SKF96365.** (**a**) Intracellular Ca^2+^ concentration ([Ca^2+^]_i_) signals induced in immobilized sperm by 3 µM progesterone (P4, black bar) followed by 30 µM SKF96365 (SKF, red bar) applied in the continued presence of P4. Representative single-cell traces are shown. (**b**) Amplitude of the transient increase in [Ca^2+^]_i_ induced by P4 (black) and by subsequent application of SKF in the continued presence of P4 (red; mean±SEM; n = 6 experiments; *P* = 0.06, paired *t*-test). (**c**) percentage of sperm in which repetitive [Ca^2+^]_i_ activity (osc) was observed following exposure to P4 (black bar) or subsequent application of SKF (red bar; n = 6 experiments; *P* = 0.00004, *t*-test). Bars in (b) and (c) show mean±SEM.

To assess the effect of SKF on P4-induced [Ca^2+^]_i_ oscillations in human sperm, we first applied P4 (3 µM), which induced a Ca^2+^ transient in 95.5 ± 1.9% of cells, followed by [Ca^2+^]_i_ oscillations in 44.7 ± 2.7% of cells (n = 10 experiments). After allowing time for completion of the P4-induced [Ca^2+^]_i_ transient and establishment of oscillations (5–10 min), we added SKF (30 µM) in the continued presence of P4. SKF failed to inhibit P4-induced [Ca^2+^]_i_ oscillations. Instead, in most cells (82.4 ± 8.4%; n = 6 experiments), 30 µM SKF induced a brief [Ca^2+^]_i_ transient, of similar amplitude to that caused by P4 (*P* = 0.06; Fig. 1a and b, Supplementary Fig. S1), and also significantly increased the proportion of cells in which [Ca^2+^]_i_ oscillations were observed (81.9 ± 3.8%; *P* = 0.00004; Fig. 1c). We therefore investigated the effects of SKF without prior exposure to P4.

### SKF increases [Ca^2+^]_i_ in human sperm

**Figure 2.**
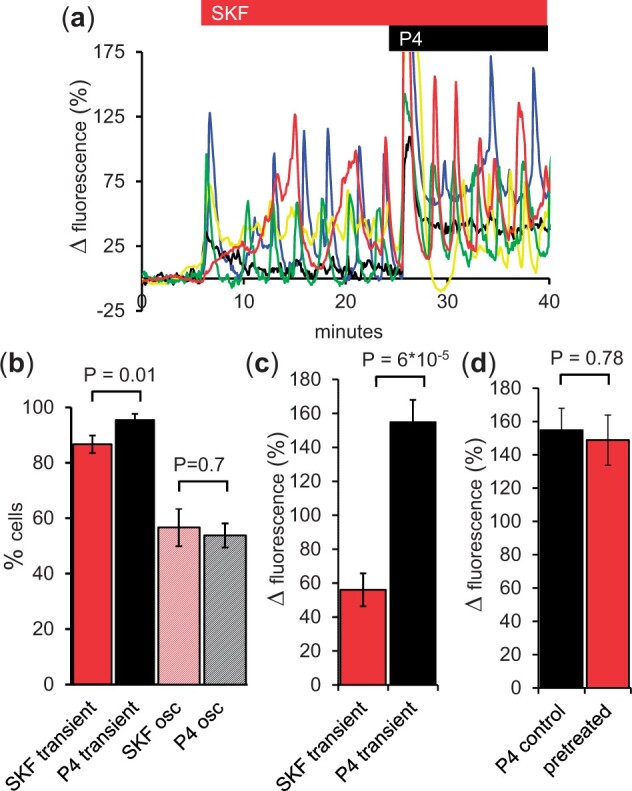
**Effect of 30 μM SKF96365 on [Ca^2+^]_i_ in human sperm.** (**a**) [Ca^2+^]_i_ signals induced by 30 µM SKF96365 (SKF, red bar) followed by 3 µM progesterone (P4, black bar) in immobilized human sperm. Representative single cell traces are shown. (**b**) percentage of cells in which an initial [Ca^2+^]_i_ response (transient) and repetitive [Ca^2+^]_i_ activity (osc) were observed in response to SKF alone (red and red crosshatched bars; n = 8 experiments) and progesterone (P4) alone (black and black crosshatched bars; n = 10 experiments). (**c**) amplitude of initial [Ca^2+^]_i_ response (transient) induced by SKF alone (red bar; n = 7 experiments) and by P4 alone (black bar; n = 11 experiments). (**d**) amplitude of initial [Ca^2+^]_i_ transient induced by P4 alone (control, black bar; n = 11 experiments) and by P4 when applied after pre-treatment with SKF (pretreated, red bar; n = 7 experiments). Bars show mean±SEM. *P* values show results of Student’s *t*-tests.

Upon application of 30 µM SKF, 87 ± 3% of cells generated an immediate [Ca^2+^]_i_ transient (1–2 min duration) or slowly developing plateau (Fig. 2a and b; n = 9 experiments, *P* = 0.01 compared to 3 µM P4), followed by oscillations in 56 ± 7% of cells (Fig. 2a and b, *P* = 0.7 compared to 3 µM P4). The initial [Ca^2+^]_i_ response evoked by SKF was clearly smaller than that seen with P4 in parallel experiments (*P* = 0.00006; Fig. 2c) and also than the [Ca^2+^]_i_ transients observed when SKF when was applied following pre-treatment with P4 (*P* = 0.003; compare responses to SKF in Figs 1b and 2c). When cells were exposed to P4 after pre-treatment with SKF, we observed a [Ca^2+^]_i_ transient of similar amplitude to that induced by P4 under control conditions (*P* = 0.78; Fig. 2a and d).

**Figure 3.**
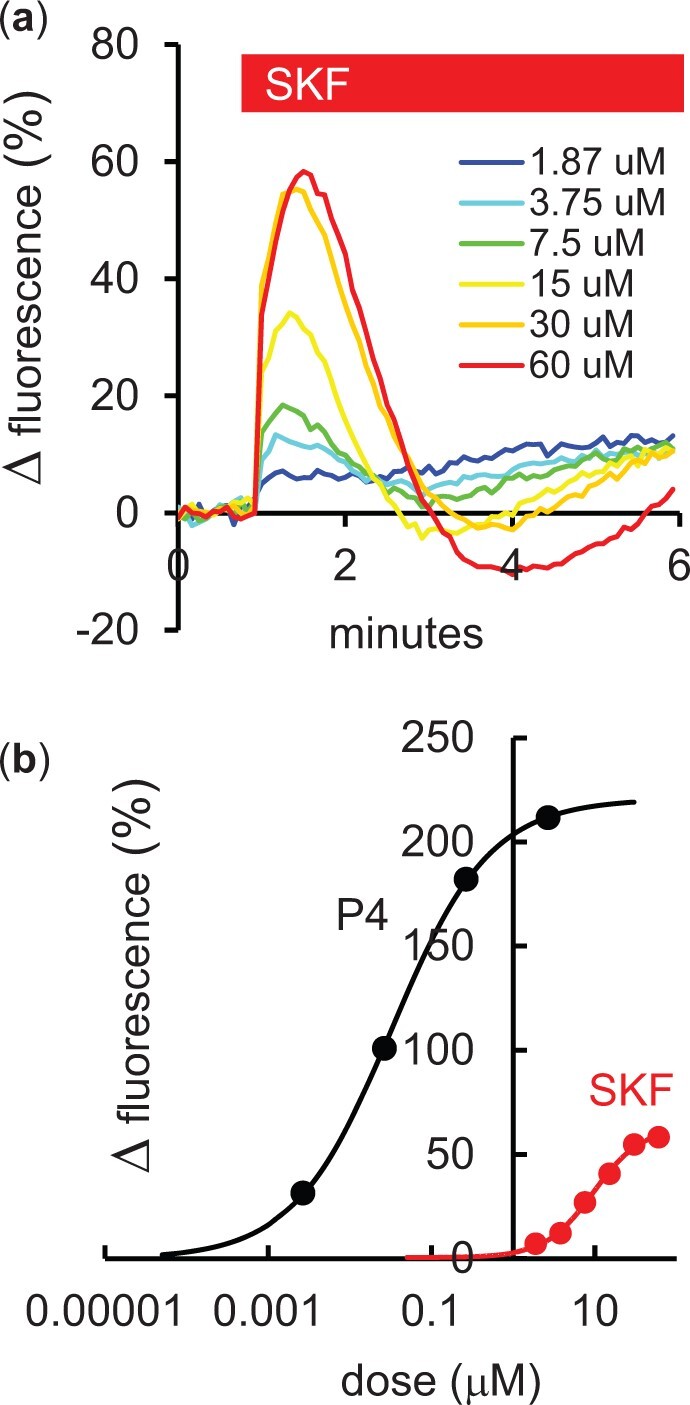
**Effect of SKF96365 on [Ca^2+^]_i_ in human sperm populations, assessed by fluorimetric recording.** (**a**) Example traces from a single experiment. Six doses of SKF96365 (SKF, 1.87–60 µM) were tested in parallel using a fluorimetric plate reader. The vehicle (dimethylsulphoxide: DMSO) at a concentration equivalent to that present in the highest dose used (0.6%) had a negligible effect (Achikanu *et al.*, 2018). (**b**) Dose-dependence of the effects of SKF (red line; mean of six experiments) and progesterone (P4; black line; mean of seven experiments). The EC_50_ values obtained from the fitted curves were 9.5 and 0.032 µM for SKF and P4, respectively.

To provide a better comparison of efficacy of the two stimuli, we used population fluorimetry to study the dose-dependence of [Ca^2+^]_i_ transient amplitudes induced by SKF and P4 (Fig. 3a and b). The EC_50_ values obtained by fitting curves to the data for SKF (n = 6 experiments) and for P4 (n = 7 experiments) were 9.5 and 0.032 µM, respectively. Furthermore, the saturating amplitude of the SKF-induced response was <30% of that for P4 (Fig. 3b). Vehicle (DMSO) at the doses used had negligible effect (Achikanu *et al.*, 2018).

### Action of SKF96365 on human sperm involves CatSper

**Figure 4.**
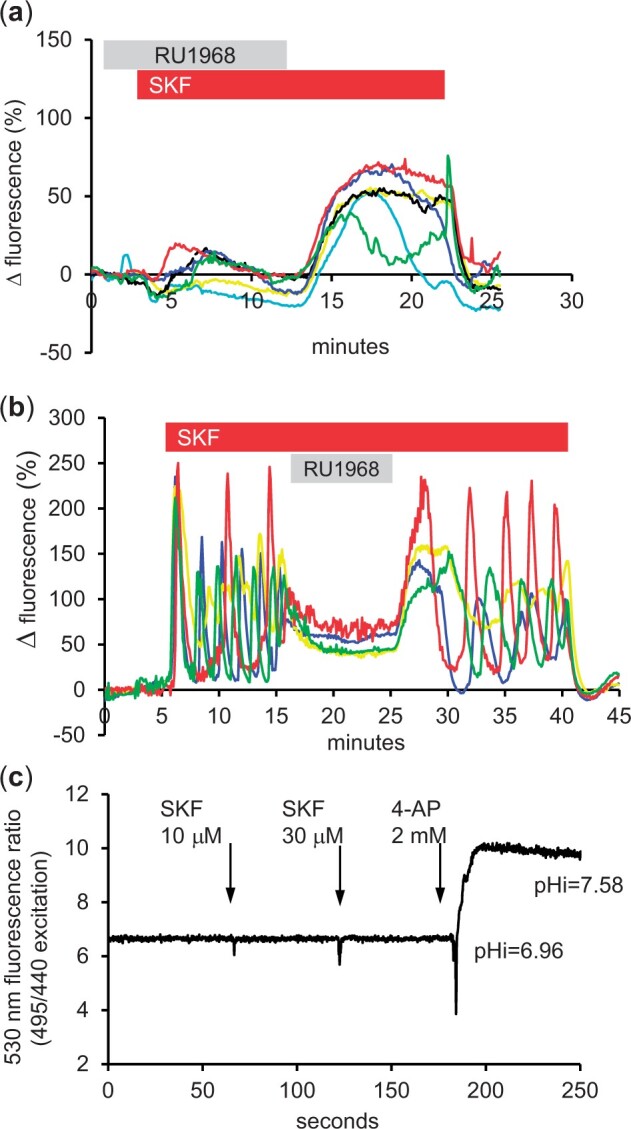
**Effects of 30 µM SKF96365 on [Ca^2+^]_i_ were inhibited by the CatSper blocker RU1968.** (**a**) Responses of immobilized sperm to treatment with 30 µM SKF96365 (SKF, red bar) in the presence of RU1968 (11 µM; Torrezan-Nitao *et al.*, 2021; grey bar). A small fraction of cells generated an initial [Ca^2+^]_i_ transient in response to SKF (e.g. red trace) but upon washout of RU1968, all cells showed a ‘rebound’ increase in [Ca^2+^]_i_, which persisted in many cells until washout of SKF. (**b**) inhibition of SKF-induced repetitive [Ca^2+^]_i_ activity (red bar shows SKF application) by exposure to 11 µM RU1968 in the superfusing saline (grey bar). When RU1968 was washed out, oscillations recovered and continued until SKF was removed. (**c**) fluorimetric assessment of pHi in a population of human sperm (monitored using 2′,7′-bis-(2-carboxyethyl)-5-(and-6)-carboxyfluorescein (BCECF)), during exposure to doses of SKF or 2 mM 4-aminopyridine (4-AP; timing of treatments shown with arrows). Neither 10 µM (first arrow) nor 30 µM (second arrow) SKF96365 caused a measurable increase in pHi but addition of 2 mM 4-AP (third arrow, positive control) caused an immediate rise of more than 0.5 units. CatSper: cation channel of sperm.

The [Ca^2+^]_i_ transient and subsequent oscillations induced by stimulation with P4 are dependent on CatSper and are strongly inhibited by the CatSper blocker RU1968 (Rennhack *et al.*, 2018; Torrezan-Nitao *et al.*, 2021). We pre-treated sperm with 30 µM RU1968 (dose applied by perfusion = 11 µM; Torrezan-Nitao *et al.*, 2021) for 2 min, then stimulated the cells with 30 µM SKF. The initial transient occurred in only 7% of cells (n = 75 cells)—(e.g. red trace in Fig. 4a) and we observed no [Ca^2+^]_i_ oscillations. However, upon washout of RU1968, we saw a rebound [Ca^2+^]_i_ elevation in all cells, which decayed upon washout of SKF (Fig. 4a). In P4-pretreated cells, established [Ca^2+^]_i_ oscillations are reversibly inhibited by RU1968 (Torrezan-Nitao *et al.*, 2021). We therefore stimulated cells with SKF to induce [Ca^2+^]_i_ oscillations, then applied 11 µM RU1968. Similarly to the effect of the drug on P4-induced oscillations, the proportion of cells in which oscillations occurred was reduced from 73.2 ± 10.4% to 5.5 ± 1.5% (*P* = 0.017; n = 3 experiments) but oscillations recovered upon washout of the CatSper blocker and persisted until washout of SKF (Fig. 4b). Since CatSper is sensitive to intracellular alkalinization, we checked whether SKF modified intracellular pH. We observed no effect of SKF, whereas 4-aminopyridine (a weak base that rapidly increases pHi in human sperm; Alasmari *et al.*, 2013) increased pHi by almost 0.6 units (Fig. 4c).

**Figure 5.**
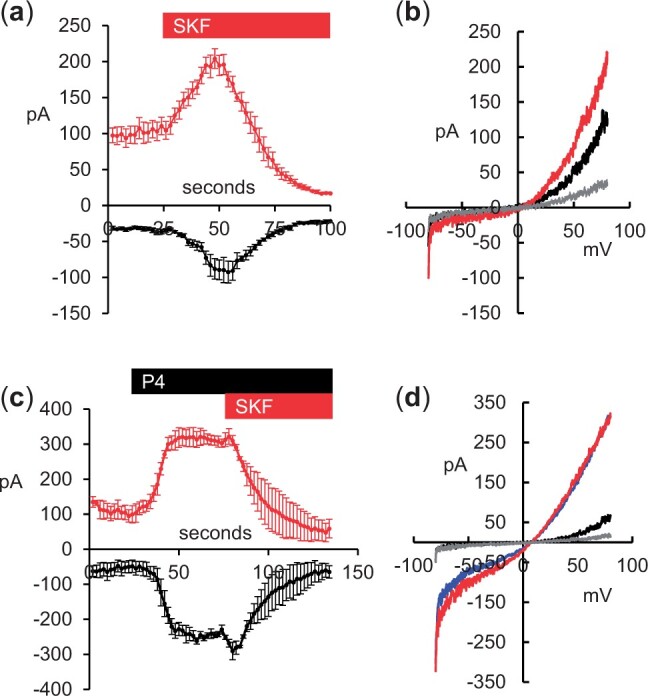
**Effects of 30 μM SKF96365 on CatSper currents.** Cs^+^ currents through cation channel of sperm (CatSper) channels were evoked by ramps (−80 to 80 mV; 1 s duration) applied continuously at 0.5 Hz. Amplitudes of the currents recorded at +80 mV (outward, red) and −80 mV (inward, black) are plotted against time. (**a**) upon application of SKF96365 (SKF, red bar), the amplitude of both inward and outward currents increased transiently then decayed over the next 30–60 s (*P* < 0.01). Points show mean±SEM of six cells. (**b**) Example traces from a cell stimulated as in panel a. Black trace shows a control current recorded before application of SKF, red trace shows maximum current, recorded 25 s after application of SKF, grey trace shows current recorded 75 s after application of SKF. (**c**) Cells were first stimulated with 3 µM progesterone (P4; black bar), which significantly increased inward and outward currents (*P* < 0.01, paired Student’s *t*-test). Exposure to SKF (red bar), in the continued presence of P4, briefly stimulated inward current, then reduced currents to levels at or below those recorded before stimulation. Points show mean±SEM of three cells. (**d**) Example traces from a cell stimulated as in panel c. Black trace shows a control current recorded at the start of the experiment, blue trace shows a current recorded in the presence of P4, red trace shows maximum current, recorded 10 s after application of SKF, grey trace shows current recorded 60 s after application of SKF.

These findings suggest that SKF can stimulate CatSper, via a pH-independent mechanism, as has been described previously for a number of small organic compounds (Brenker *et al.*, 2012; Tavares *et al.*, 2013; Schiffer *et al.*, 2014; Birch *et al.*, 2022). We therefore used patch clamp directly to measure the effect of SKF treatment on monovalent (Cs^+^) CatSper currents. Consistent with the effects of SKF on [Ca^2+^]_i_, CatSper currents increased ‘immediately’ upon application of SKF, reaching a maximum after 20–30 s (*P* = 0.024; n = 6 cells; Fig. 5a and b). However, current amplitude then decreased over the next 30–60 s, falling to values below pre-stimulus levels (*P* = 0.01; Fig. 5a and b). When cells were exposed to P4 (3 µM), inward and outward currents rapidly increased, reaching a plateau within 30 s (*P* < 0.01; n = 3 cells; Fig. 5c and d). Subsequent application of SKF caused a brief increase in current amplitude (more clearly visible in inward than outward currents), but currents then decreased over the next 40–50 s, reaching levels at or below those recorded before stimulation (Fig. 5c and d).

### Capacitation and the action of SKF

**Figure 6.**
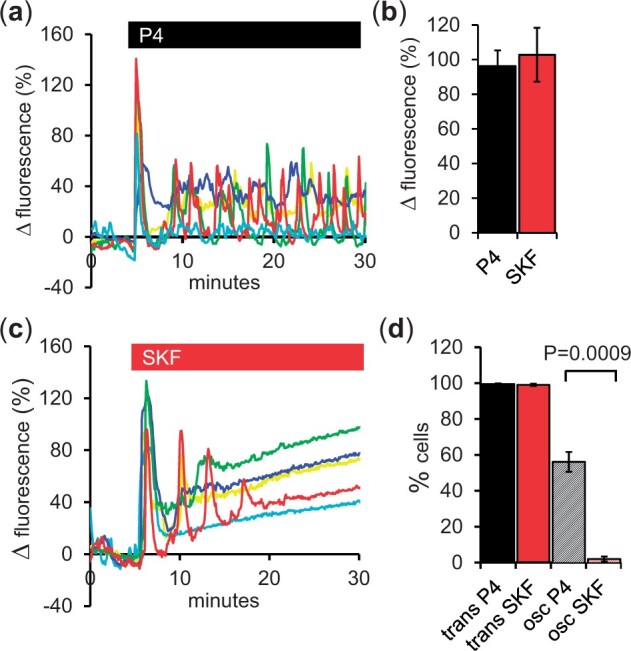
**Effects of SKF96365 were dependent upon presence of bovine serum albumin.** (**a**) Responses of immobilized individual sperm to 3 µM progesterone (P4, black bar) in bovine serum albumin (BSA)-free medium. All these cells generated an initial [Ca^2+^]_i_ transient and in four cells this was followed by repetitive [Ca^2+^]_i_ activity/oscillations that persisted until the end of the recording (25 min later). (**b**) Amplitude of the initial [Ca^2+^]_i_ transient in sperm prepared in BSA-free medium is similar in cells stimulated with progesterone (P4, black bar) and with SKF96365 (SKF, red bar; n = 5 experiments for each treatment). (**c**) Responses of individual sperm to 30 µM SKF (red bar) in BSA-free medium. All these cells generated an initial [Ca^2+^]_i_ transient but only one cell (red trace) generated repetitive [Ca^2+^]_i_ activity, which decayed and arrested after three cycles. (**d**) Percentage of cells showing a transient [Ca^2+^]_i_ response (solid bars) and subsequent repetitive [Ca^2+^]_i_ activity/oscillations (crosshatched bars) following treatment with 3 µM P4 (black bars) or 30 µM SKF (red bars; *P* = 0.0009; Student’s *t*-test, n = 5 experiments for each treatment). Bars show mean±SEM.

To further compare the abilities of P4 and SKF to induce oscillations, we investigated the dependence of oscillations on prior incubation of the cells in capacitating medium. We therefore carried out a series of experiments using a modified medium from which BSA was omitted, a procedure which impairs cytoplasmic alkalinization during capacitation of human sperm (Zhao *et al.*, 2021). As reported previously (Bedu-Addo *et al.*, 2005), this procedure did not abolish the P4-induced [Ca^2+^]_i_ transient (present in 99% of cells), but significantly reduced its amplitude from 155 ± 13% under standard conditions (Fig. 1b) to 96 ± 10% (Fig. 6a and b; *P* = 0.025). [Ca^2+^]_i_ oscillations occurred in 56 ± 5% of cells (Fig. 6a and d). However, in cells stimulated with SKF, >95% responded to the drug with an initial [Ca^2+^]_i_ transient, which was similar in amplitude to that seen with P4 (number ± SD; Fig. 6b and c), but only 24.3 ± 6.8 generated any repetitive activity (typically ≤3 cycles, e.g. Fig. 6c, red trace) and just 2.3 ± 1.4% generated sustained, repetitive activity similar to that seen with P4 (Fig. 6d;*P* = 0.0009 compared to P4). Instead, the [Ca^2+^]_i_ transient was typically followed by a slowly rising plateau (Fig. 6c).

## Discussion

SKF, a commonly used blocker of CCE (Labelle *et al.*, 2007; Ng *et al.*, 2008, 2010; Li *et al.*, 2010; Sabourin *et al.*, 2016), which can also inhibit other Ca^2+^-permeable channels (Varnai *et al.*, 2009), might be expected to suppress [Ca^2+^]_i_ signalling in human sperm. Instead, we observed a dose-dependent increase in [Ca^2+^]_i_ upon SKF-treatment. This effect of SKF resembled the well-characterized action of P4 on human sperm in that: it induced a transient increase in [Ca^2+^]_i_ in >80% of cells (though the potency of SKF was significantly lower than P4; Fig. 3b); following the [Ca^2+^]_i_ transient, oscillations, or irregular [Ca^2+^]_i_ spiking occurred in 50% of cells; and both the [Ca^2+^]_i_ oscillations and the preceding [Ca^2+^]_i_ transient were sensitive to the CatSper blocker RU1968. Though unexpected, these observations are perhaps not surprising. CatSper is known to be highly promiscuous, being activated by a wide variety of small organic molecules including a range of pharmacological tools, insecticides, and sunscreens (Brenker *et al.*, 2012; Tavares *et al.*, 2013; Schiffer *et al.*, 2014; Birch *et al.*, 2022). It is also possible that the SKF-induced [Ca^2+^]_i_ transient involves acrosomal alkalinization and consequent mobilization of stored Ca^2+^. This effect has been observed in human sperm treated with some other Ca^2+^ channel blockers, particularly NNC55-0396 (Chavez *et al.*, 2018). However, if such an effect occurs with SKF, its contribution to the observed elevation of [Ca^2+^]_i_ is likely to be small, since the effect of SKF was virtually abolished by pre-treatment with RU1968.

Unlike many of these agents, the efficacy of SKF was enhanced rather than occluded by prior exposure to P4, and pre-treatment with SKF had no effect on the response to P4. This indicates that SKF and P4 activate CatSper through independent mechanisms. Since SKF did not raise pHi (indirectly activating CatSper), it appears that SKF may activate CatSper ‘directly’ but by a different mechanism to P4, as is the case for activation by prostaglandins (Schaefer *et al.*, 1998; Lishko *et al.*, 2011; Strunker *et al.*, 2011). Future studies should address whether the action of SKF is sensitive to prior activation of CatSper by stimulation with prostaglandin.

Consistent with this interpretation of the action of SKF, assessment of CatSper activity by patch clamp showed that SKF, like P4, caused an immediate increase in current amplitude (Lishko *et al.*, 2011; Fig. 6a). However, unlike the effect of P4, in the presence of SKF the amplitudes of both inward and outward currents then fell to levels below those observed under control conditions, even in cells where CatSper channels had previously been stimulated with P4 (Fig. 6b). This reversal of the tonic stimulation seen with P4 indicates the occurrence of a second, inhibitory action of SKF, rather than a simple decay of the stimulatory effects. SKF is not considered to be specific to CCE in its inhibitory actions, having effects on other Ca^2+^ channel types including receptor-operated and voltage-operated Ca^2+^ channels (VOCCs; Varnai *et al.*, 2009). In particular, similarly to some previously characterised blockers of CatSper (e.g. Ni^2+^, NNC55–0396, Mibefradil; Lishko *et al.*, 2011; Strunker *et al.*, 2011), SKF has been shown to be a blocker of T-type VOCCs (Singh *et al.*, 2010).

Dual actions of drugs (e.g. RU1968, sertraline) on [Ca^2+^]_i_ in human sperm, apparently reflecting an initial stimulation of CatSper, lasting a few minutes, followed by a tonic inhibitory effect, have been described previously (Rennhack *et al.*, 2018; Rahban *et al.*, 2021). The transient nature of the increase in CatSper current (60–100 s; Fig. 6a) is consistent with such observations and apparently provides an explanation for the similarly brief increase in [Ca^2+^]_i_ seen in SKF stimulated human sperm. However, other observations cannot be reconciled with this interpretation. Firstly, in cells previously exposed to P4, [Ca^2+^]_i_ oscillations (which require CatSper activity; Torrezan-Nitao *et al.*, 2021) persisted for at least 10 min in the presence of SKF (often being enhanced; Fig. 1a). More strikingly, when the cells were pre-treated with SKF, subsequent application of P4 (in the continued presence of SKF) was able to induce a [Ca^2+^]_i_ transient (known to be dependent on CatSper channels; Strunker *et al.*, 2011) of similar amplitude to that seen in parallel control experiments (Fig. 2d). Thus, it appears that the delayed inhibition of CatSper by SKF seen in our patch clamp experiments does not occur in imaging experiments on intact cells. We are currently unable to provide an explanation for these observations. One possibility is that the blocking action of SKF is use- or voltage-dependent, such that this effect is revealed only by the repeated manipulations used in our patch clamp experiments. Future studies should address these questions by varying patch clamp protocols to test use-dependence (e.g. dependence of current decay on frequency of stimulation) and open/closed sensitivity (e.g. by varying the holding voltage of the cell during SKF pre-treatment). A further complication is that treatment with SKF96365 (5–20 µM) inhibited motility in human sperm, consistent with an inhibitory action on CatSper and/or CCE (Castellano *et al.*, 2003), yet we observed an increase of [Ca^2+^]_i_ in intact cells. Regardless of the underlying mechanism, these observations suggest that patch clamp recordings of CatSper and possibly other channels in human sperm may not always reflect the behaviour of channels in intact sperm and should be interpreted cautiously.

To investigate the significance of capacitation for the action of SKF, we assessed the effects of SKF and P4 in cells where capacitation was impaired by the absence of BSA. BSA has been shown to act as an agonist of the proton channel Hv1 in human sperm and the capacitation-associated rise in pHi (and consequent increased sensitivity of CatSper to activating stimuli) is BSA-dependent (Zhao *et al.*, 2021). We found that, in the absence of BSA, both P4 and SKF were still able to induce a [Ca^2+^]_i_ transient in a large majority of cells, but their ability to support subsequent repetitive activity (oscillations) was markedly different. Whereas P4 induced oscillations in more than 50% of cells, SKF-generated, repetitive activity was very rare. This suggests that treatment with P4 may ameliorate the effects of BSA-free incubation, perhaps by activation of other signalling events, as has been described previously in human and primate sperm (e.g. Thomas and Meizel, 1989; Sagare-Patil *et al.*, 2012; Sagare-Patil and Modi, 2013; Sumigama *et al.*, 2015) leading to sensitization of CatSper. Significantly, we have also observed that stimulation with prostaglandin E_1_, which activates CatSper by a different mechanism to P4, can induce [Ca^2+^]_i_ oscillations both in ‘capacitated’ sperm and in cells prepared in the absence of BSA (Torrezan-Nitao, unpublished data).

In summary, the effects of SKF on Ca^2+^ signalling in human sperm are complex. The drug both stimulates and inhibits currents through CatSper channels, though the inhibitory effect is not apparent in the actions of SKF on [Ca^2+^]_i_ in imaged cells. These complex effects preclude the use of SKF to investigate involvement of CCE in human sperm Ca^2+^ signalling.

## Supplementary Material

gaad015_Supplementary_Data

## Data Availability

The data underlying this article will be shared on reasonable request to the corresponding author.
