# Supplementary material for: SKF96365 modulates activity of CatSper channels in human sperm
Source: Mol Hum Reprod. 2023 Apr 27;29(6):gaad015. doi: 10.1093/molehr/gaad015 (PMC10266451; doi:10.1093/molehr/gaad015)
Supplement: gaad015_Supplementary_Data [file gaad015_supplementary_data.pdf]

Supplementary Information

SKF96365 modulates activity of CatSper channels in human sperm.

Elis Torrezan-Nitao, Sean Brown, Linda Lefievre, Jennifer Morris, Joao Correia, Claire Harper, Stephen Publicover.

Supplementary Figure S1. Pre-treatment of human sperm with 3 µM progesterone does not occlude the response to 30 µM SKF96365.

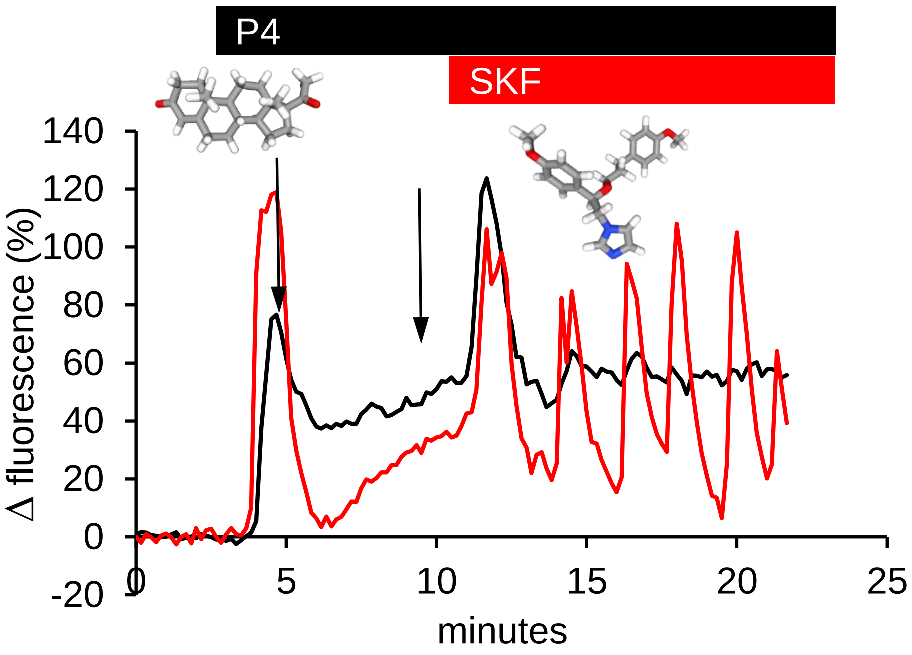

Supplementary Figure S1. Pre-treatment of human sperm with 3 μM progesterone does not occlude the response to 30 μM SKF96365.

Plot shows an example of a mean $[Ca^{2+}]_i$ trace (mean of all cells in an experiment; black line) and a single cell $[Ca^{2+}]_i$ trace from the same experiment (red line) in which stimulation with 3 μM progesterone (P4, black bar) was followed by 30 μM SKF96365 (SKF, red bar). P4 failed to induce oscillations in the cell shown here, but oscillations occurred after stimulation with SKF. Both traces clearly show the stimulatory effect of SKF in progesterone pre-treated cells, the response to SKF being of similar amplitude to that induced by the preceding application of P4. Molecular structures for progesterone (left) and SKF96365 (right), shown above the traces, are from Pubchem (https://pubchem.ncbi.nlm.nih.gov/).
